# Development and usability of the MAINtAIN, an inventory assessing nursing staff behavior to optimize and maintain functional activity among nursing home residents: a mixed-methods approach

**DOI:** 10.1186/s12913-016-1288-7

**Published:** 2016-02-02

**Authors:** Nienke O. Kuk, G. A. Rixt Zijlstra, Gerrie J. J. W. Bours, Jan P. H. Hamers, Gertrudis I. J. M. Kempen

**Affiliations:** 1 Department of Health Services Research, CAPHRI School for Public Health and Primary Care, Maastricht University, Maastricht, The Netherlands; 2 Zuyd University of Applied Sciences, Research Centre for Autonomy and Participation for Persons with a Chronic Illness, Heerlen, The Netherlands

**Keywords:** Activities of daily living, Barriers, Behavior, Development, Facilitators, Usability studies, Functional activity, Nursing, Nursing homes, Questionnaires

## Abstract

**Background:**

Functional decline is common in nursing home residents. Nursing staff can help prevent this decline, by encouraging residents to be more active in functional activities. Questionnaires measuring the extent to which nursing staff encourage functional activity among residents are lacking. In addition, there are no measurement instruments to gain insight into nursing staff perceived barriers and facilitators to this behavior. The aim of this study was to develop, and study the usability, of the *MA*astr*I*cht *N*urses *A*ctivities *IN*ventory (MAINtAIN), an inventory assessing a) the extent to which nursing staff perceive to perform behaviors that optimize and maintain functional activity among nursing home residents and b) the perceived barriers and facilitators related to this behavior.

**Methods:**

Using a mixed-methods approach the MAINtAIN was developed and its usability was studied. Development was based on literature, expert opinions, focus group (*N* = 3) and individual interviews (*N* = 14) with residents and staff from nine nursing homes in the Netherlands. Usability was studied in a cross-sectional study with 37 nurses and certified nurse assistants; data were analyzed using descriptive statistics.

**Results:**

Development of the MAINtAIN resulted in two distinctive parts: MAINtAIN-behaviors and MAINtAIN-barriers. MAINtAIN-behaviors, targeting nursing staff behavior to optimize and maintain functional activity, includes 19 items covering activities of daily living, household activities, and miscellaneous activities. MAINtAIN-barriers addresses the perceived barriers and facilitators related to this behavior and comprises 33 items covering barriers and facilitators related to the residents, the professionals, the social context, and the organizational and economic context. The usability study showed that the inventory was not difficult to complete, that items and response options were clear, and that the number of missing values was low. Few items showed a floor or ceiling effect.

**Conclusions:**

The newly developed inventory MAINtAIN provides a usable method for researchers and nursing homes to obtain insight into nursing staff perceived behavior in optimizing functional activity among residents and their perceived barriers and facilitators related to this behavior. Outcomes of the MAINtAIN may contribute to change in nursing staff behavior and may improve nursing care. Further research with regard to the psychometric properties of the MAINtAIN is recommended.

**Electronic supplementary material:**

The online version of this article (doi:10.1186/s12913-016-1288-7) contains supplementary material, which is available to authorized users.

## Background

With increasing age, the risk of functional decline increases. This is one of the major risk factors for older people to transfer to a nursing home [1, 2]. Once residing in a nursing home, individuals often have a low activity level [3–6] and further functional decline is common [7, 8]. However, the functional level of nursing home residents does not, by definition, have to decline [9]. To maintain their functional level it may be important that nursing home staff encourage residents to perform functional activities and stay as active as possible [10, 11].

Although nursing staff are not the only ones who can play a role, it is particularly relevant for them to provide care that optimizes the functional level of residents as they are generally the professionals that spend time with residents during moments and activities that allow residents’ physical engagement. They can encourage residents and provide them with opportunities to be active during daily care activities, instead of taking over the care. However, nursing staff may experience certain barriers that might make it difficult for them to provide the best possible care with regard to optimizing functional activity among nursing home residents [12]. Identifying these barriers, or in contrast facilitators, is an important step towards improving care [13].

Before nursing care can be improved, it is essential to gain insight into the care nursing staff currently provide. Although studies measuring low activity levels of residents [3–5] indicate that nursing staff could encourage residents more often to be active, no data are available that provide insight into the extent to which and during what activities nursing staff think they encourage the functional abilities of residents. To our knowledge, questionnaires addressing this issue are lacking, as well as questionnaires explicitly focusing on nursing staff perceived barriers or facilitators related to optimizing and maintaining functional activity among nursing home residents. Such questionnaires could provide a national overview of the perceived behaviors, barriers and facilitators of nursing staff or could be used as a baseline measure for quality improvement projects in individual nursing homes.

The aim of this study is to develop a usable inventory assessing a) the extent to which nursing staff perceive to perform behavior to optimize and maintain functional activity among nursing home residents and b) the perceived barriers and facilitators related to this behavior. This article describes the process of developing, and studying the usability of, the MAastrIcht Nurses Activities INventory (MAINtAIN).

## Methods

**Fig. 1:**
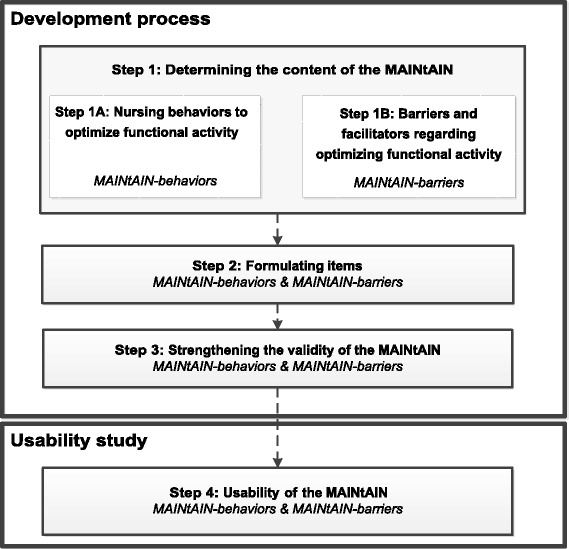
Development process of the inventory: a four-step approach. Legend: MAINtAIN: MAastrIcht Nurses Activities INventory

To develop and study the usability of the MAINtAIN a mixed-methods approach was used, involving a multidisciplinary group of stakeholders. The process consisted of four steps (see Fig. 1). The results of each step guided the following steps. The MAINtAIN is developed to comprise two parts. Part one aims to assess the behaviors nursing staff perceive to perform during their daily care to optimize and maintain functional activity among residents (MAINtAIN-behaviors). Part two aims to address barriers and facilitators nursing staff perceive when optimizing functional activity among residents (MAINtAIN-barriers). With regard to nursing behaviors, the focus is on behaviors that can be performed by nursing staff during daily nursing care. In this article, the term “nursing behaviors” is used; however, the inventory is also intended for certified nurse assistants (CNAs) or other direct-care staff. The study was approved by the Medical Ethical Review Committee of Maastricht University (12-5-062).

### Development process

#### Step 1: Determining the content of the MAINtAIN

In the first step, the content of MAINtAIN-behaviors and MAINtAIN-barriers was determined in two separate, parallel processes, as is shown in Fig. 1. For MAINtAIN-behaviors, data from the literature and experts were retrieved. For MAINtAIN-barriers, literature was reviewed and nursing home professionals and residents were consulted.

##### Step 1A: Determining content of MAINtAIN-behaviors: nursing behaviors to optimize and maintain functional activity

Based on previous literature, a list of nursing behaviors that may lead to an improvement in functional activity among nursing home residents was formulated. Nursing behaviors focusing on activities that could be incorporated into daily nursing home care were taken into consideration. Sources were retrieved from the PubMed, CINAHL and Google Scholar databases. Search terms included ‘nurses’, ‘behaviors’, ‘interventions’, ‘function’, ‘functional status’, ‘nursing home residents’, ‘activities of daily living’, and ‘older people’. In addition, snowball search techniques were used, meaning the reference lists of relevant articles were searched for new relevant literature. Furthermore, a search for specific interventions in evidence-based guidelines and protocols, and textbooks was performed.

To determine which of the listed nursing behaviors were relevant for the inventory, the list was sent to 12 experts, working in the field of elderly care or with a nursing background. These experts worked at different departments of Maastricht University (*n* = 9), at Zuyd University of Applied Sciences (*n* = 2), and at a nursing home (*n* = 1) in the south of the Netherlands. Eight of these experts were or had been active as registered nurse (RN). The experts were asked to rank the listed behaviors according to their feasibility and relevance in contributing to the functional activity of nursing home residents. Extra behaviors deemed important could be added. Based on these rankings and the comments of the experts, authors NOK, GARZ, and GJJWB made a selection of behaviors to be incorporated in MAINtAIN-behaviors.

##### Step 1B: Determining the content of MAINtAIN-barriers: barriers and facilitators regarding optimizing and maintaining function

First, to gain insight into existing knowledge and identify key articles on nursing staff perceived barriers and facilitators to optimizing and maintaining functional activity among nursing home residents a general literature search was performed in different databases (PubMed, CINAHL, Google Scholar). In addition, these databases were searched for existing questionnaires measuring such barriers or facilitators. Search terms included ‘function’, ‘functional status’, ‘barriers’, ‘facilitators’, ‘nursing home’, ‘older persons’, and ‘nurses’. Although the primary interest was in questionnaires assessing barriers or facilitators specifically related to optimizing functional activity, questionnaires measuring barriers and facilitators in general were also taken into account. Snowball search techniques were used as well.

Second, to ensure that MAINtAIN-barriers comprised issues residents and nursing home professionals encounter, focus group interviews and individual interviews were conducted. Residents and staff were recruited from nine nursing homes of two large long-term care organizations in the south of the Netherlands. Although nursing staff will be the end users of the inventory, a rich information base was ensured by recruiting a heterogeneous sample of participants, based on educational backgrounds and professions.

To begin, three focus group interviews were conducted. To promote an environment in which participants felt comfortable talking [14], homogeneity within groups was created based on similarities in educational level. The first group consisted of a nursing home manager (*n* = 1), ward managers (*n* = 2), physiotherapists (*n* = 3), and a nurse practitioner (*n* = 1). The second group consisted of CNAs^1^ (*n* = 2), RNs with 4 years of secondary vocational training (*n* = 3), and a bachelor’s prepared RN (*n* = 1). The third group consisted of a CNA (*n* = 1) and RNs with 4 years of secondary vocational training (*n* = 3). A topic list was used to guide the focus groups. At the end of each session, barriers and facilitators to optimizing functional activity among nursing home residents were grouped according to different levels based on work by Grol and Wensing [13]: the innovation (in this case interpreted as the functional activity improving behaviors themselves), the residents, the professionals, the social context, the organizational context, and the economic and political context.

Subsequently, 14 semi-structured individual interviews were conducted with people not participating in the focus groups, i.e., RNs with 4 years of secondary vocational training (*n* = 3), CNAs (*n* = 3), a bachelor’s prepared RN (*n* = 1), a nursing home physician (*n* = 1), a physiotherapist (*n* = 1), a location manager (*n* = 1), a policy officer (*n* = 1), and residents (individually or together with their informal caregiver) (*n* = 3). The individual interviews included topics that were also discussed in the focus group interviews.

All interviews were audio-recorded (after the participants gave written informed consent), transcribed and analyzed by author NOK. First, all relevant text was highlighted. Next, the content of the interviews was coded. A directed content analysis approach [15] was used to categorize coded barriers and facilitators according to predetermined levels. These levels are described by Grol and Wensing [13] (see above).

#### Step 2: Formulating items

Based on the data collected in Step 1, author NOK formulated preliminary items for both sections of the MAINtAIN. Multiple items covering the same barriers or facilitators were formulated in order to select the most appropriate items in the next step. Two experts on questionnaire development were consulted regarding methods to prevent social desirability bias when formulating items.

#### Step 3: Strengthening the validity of the MAINtAIN

To ensure content validity, the initial versions of the inventory were presented to experts in different disciplines. First, while thinking aloud, a CNA completed the inventory in the presence of author NOK. Ambiguous items were pointed out and difficulties and possible solutions were discussed. In addition, for items measuring the same concept, author NOK and the CNA discussed which option was most clear. Furthermore, the suitability of the response options was evaluated, by discussing which, and why these, response options were used by the CNA. Consequently, the inventory was revised where necessary. Next, authors NOK, GARZ, and GJJWB independently checked if the formulated items still corresponded with the concepts they were intended to measure. Differences in opinion were discussed until consensus was reached. After revisions, the concept version of the inventory was presented to experts in research and practice related to older persons or implementation science (*n* = 9). The experts individually appraised the inventory for missing or redundant items and checked if the items covered the aim of the inventory. In addition, they appraised the order and wording of the items, and the layout of the inventory. Afterwards, the inventory was revised according to their feedback, resulting in a version of the inventory used to study the usability.

### Usability study

#### Step 4: Usability of the MAINtAIN

In a cross-sectional study, the usability of the MAINtAIN was studied. The convenience sample included 37 staff nurses working on somatic wards, psychogeriatric wards or rehabilitation wards of two large long-term care organizations. Rehabilitation wards were included as it was expected that nursing staff working there were more inclined to encourage functional activities. All respondents were asked to anonymously complete the inventory.

To evaluate the usability of the inventory, 1-item questions were added regarding the time respondents needed to complete the inventory, the clarity of the domains and items, the clarity of the response options, the difficulty of the items in the inventory, and suggestions for improvement. Additionally, to gain more in-depth insight into possible difficulties regarding the completion of the inventory, author NOK interviewed a convenience sample of three respondents from one nursing home after they completed the inventory.

#### Data analyses

Statistical analyses were performed using SPSS 21 for Windows. Data were analyzed using descriptive statistics. Items were checked for missing values, and floor and ceiling effects were assessed. If individual items had a response of >50 % for the lowest or highest response option, items were examined more closely. More specifically, for these items it was checked if this effect held true for the general sample, or only for a subgroup that was expected to know more about the encouragement of functional activity. Subgroups were respondents that were mobility-prone (i.e., those working at a rehabilitation ward and those specialized in mobility) versus those that were expected to be not, the non-mobility group (i.e., the remainder of the respondents). In addition, to check the discriminative ability of the inventory, bar charts were produced separately for the general population and the subgroups. Subsequently, based on floor and ceiling effects or bar charts, authors NOK, GARZ, and GJJWB judged and discussed whether or not items should be removed or changed. The outcomes of the usability study resulted in the final version of the MAINtAIN.

## Results

### Development process

#### Step 1: Determining the content of the MAINtAIN

##### Step 1A: Determining the content of MAINtAIN-behaviors: nursing behaviors to optimize and maintain functional activity

The literature search yielded four key sources that were used to formulate a list of nursing behaviors that could potentially optimize functional activity [16–19]. Listed behaviors focused on encouraging nursing home residents to perform activities of daily living (ADL; e.g., dressing or washing) as independently as possible [16–18], for example, by providing assistive devices [16, 17], but also on more general activities (e.g., encouraging residents to be physically active, helping residents maintain their daily routines, and educating informal caregivers about the importance of functional independence [19]). Although the literature primarily focused on the independent performance of ADL, additional nursing behaviors that encouraged the performance of instrumental activities of daily living, in particular household activities (e.g., making beds, setting and clearing the table, preparing small meals), were also included.

Overall, expert’s ranking of the nursing behaviors showed that they deemed the behaviors related to household activities as particularly important opportunities to optimize functional activity. Most nursing behaviors related to general activities (grouped into the category ‘miscellaneous activities’) were deemed relevant as well. Some behaviors were perceived as redundant, not applicable to psychogeriatric residents, or not suitable in all nursing homes, and therefore the removal of these behaviors was advised (for example: ‘encourage residents to buy groceries’). The final selection of the ADL-related nursing behaviors included behaviors that encouraged independence in eating, dressing, moving about the ward, and personal hygiene. The behaviors covering household activities included the encouragement of small activities that could be performed on the ward, for example, preparing sandwiches. The miscellaneous activities were a diverse selection ranging from ‘encouraging informal caregivers not to take over activities’ to ‘discussing activities from the past and trying to maintain them’.

##### Step 1B: Determining the content of MAINtAIN-barriers: barriers and facilitators regarding optimizing and maintaining functional activity

The search in the scientific databases resulted in four main articles that provided insight into possible barriers and facilitators towards optimizing function [12, 20–22]. The search did not yield an existing questionnaire that measures barriers or facilitators to optimizing functional activity among nursing home residents. However, studies were found that qualitatively measured barriers and facilitators to restorative care, an approach to optimize function [12, 20, 21]. These studies focused on barriers and facilitators as perceived by nursing assistants. Barriers identified included refusal by residents, the lack of nursing support, and the assumption that residents could not perform activities [12, 20, 21]. In addition, barriers mentioned in questionnaires generally measuring barriers and facilitators provided input for the inventory. The Measurement Instrument Determinants of Innovations [22] provided barriers that seemed relevant, despite the fact that this questionnaire was not tailored to the nursing home setting or optimizing functional activity.

Next, focus group and individual interviews provided insight into barriers and facilitators specifically related to optimizing functional activity among nursing home residents, as experienced by nursing staff or residents on a daily basis. Analysis of the relevant data showed that the barriers and facilitators were related to all pre-determined levels; three were related to the innovation, eight to the residents and informal caregivers, eight to the professionals, nine the social context, and eleven the organizational and economic context. The exposed barriers and facilitators included, for example, the attitudes of residents, low priority, the support of a manager, and courses provided.

#### Step 2: Formulating items

Based on the outcomes of Step 1, items were formulated for both parts of the inventory. In MAINtAIN-behaviors, comprising 25 items regarding the behaviors nursing staff can perform, a distinction was made between nursing behaviors related to ADL, household activities, and miscellaneous activities. To reduce the risk of socially desirable answers, experts (*N* = 2) recommended the formulation of items on a less individual level, i.e., instead of stating ‘I encourage…’ the items stated ‘On my ward, *we encourage* residents to help set and clear the table’, or ‘On my ward, *we* compliment residents when they dress and undress themselves.’ In MAINtAIN-barriers, comprising 62 items regarding perceived barriers and facilitators, a distinction was made between barriers and facilitators related to residents (e.g., ‘Residents are afraid to walk on their own’), to professionals (e.g., ‘It is primarily the responsibility of the physiotherapist/occupational therapist to encourage residents to perform activities’), to the social context (e.g., ‘My colleagues expect me to encourage residents to help carry out household activities’), and to the organizational and economic context (e.g., ‘In my organization, encouraging physical activity is a high priority’). For practical reasons, barriers related to the innovation were grouped with those related to the residents.

#### Step 3: Strengthening the validity of the MAINtAIN

The interview with the CNA led to a change in the wording of some of the items and a reduction of the number of items. Two redundant items from MAINtAIN-behaviors and 23 redundant items from MAINtAIN-barriers were removed, resulting in 23 and 39 remaining items, respectively. Regarding the response options, the remarks of the CNA revealed that she was reluctant to use the extremes of the scale. To avoid ‘end-aversion bias’ [23], it was therefore decided to add extra response options, resulting in a change from a five- to a nine-point scale. Next, wording for some items was changed following the check by authors NOK, GARZ, and GJJWB regarding the correspondence of the items with the concepts they intended to measure. In addition, some items measuring the same barriers or facilitators were merged. Lastly, experts (*N* = 9) reviewed the content and layout of the inventory and generally agreed with both. Their feedback led to minor changes in the formulation of items to prevent ambiguous phrasing and acquiescence bias, the tendency of respondents to respond to all items in the same way. Some positively phrased items were changed into negatively phrased items and vice versa. The revisions resulted in an inventory comprised of a total of 58 items: 22 in MAINtAIN-behaviors and 36 in MAINtAIN-barriers.

### Usability study

#### Step 4: Usability of the MAINtAIN

Of the 37 respondents that completed the inventory, 13 worked in somatic wards, 10 in psychogeriatric wards, and 14 in rehabilitation wards. The majority were CNAs (86.5 %). Table 1 describes the characteristics of the study population.

**Table 1:** Study population characteristics (*N* = 37)

	Number	Percent
Gender		
Female	32	86.5
Ward		
Somatic	13	35.1
Psychogeriatric	10	27.0
Rehabilitation	14	37.8
Profession		
Certified nurse assistants	29	78.4
Registered nurses with 4 years of secondary vocational training	7	18.9
Bachelor’s prepared registered nurses	1	2.7
	Mean	SD
Age (years)	38.2	12.2
Years working in geriatric care	14.3	9.5
Contract hours per week	29.1	6.2

The mean response rate per item was 98.9 % (ranging from 91.9 to 100 %). The mean time required to complete the full inventory was 14 min (range 7–30 min). Respondents indicated that the domains and items were clear and that the inventory was not difficult. In addition, respondents, including the interviewed respondents, unanimously answered that the response options were clear. The interviewed respondents provided only minor suggestions regarding the wording of the items.

Analyses revealed that five of the 58 items showed a floor- or ceiling effect, i.e., one of the extreme response options was chosen by >50 % of the respondents (see Table 2). As Table 2 shows, these floor and ceiling effects were mainly present in the mobility-prone group. After further discussion, the researchers decided to remove one of these five items, i.e., an item regarding instructing residents how to use walking aids. The researchers agreed that a broader item, encouraging residents to move about independently, covered this item. The other four items were retained for theoretical reasons. Visual inspection of bar charts, in particular for the items of MAINtAIN-behaviors, indicated that the inventory distinguished between the mobility-prone group and the non-mobility group.

**Table 2:** Inventory items that showed a floor or ceiling effect in the study population

Item	Extreme value	Total (*N* = 37)		Mobility-prone group^b^ (*N* = 16)		Non-mobility group^c^ (*N* = 21)	
		n	(%)	n	(%)	n	(%)
MAINtAIN-behaviors							
It is not relevant for residents on my ward to independently perform ADL (such as bathing and dressing).	Completely disagree^d^	20	(55.6)^a^	10	(66.7)^a^	10	(47.6)
MAINtAIN-barriers							
On my ward…							
We compliment residents when they manage to dress and undress themselves.	Always^e^	20^f^	(54.1)^f,^ ^a^	10^f^	(62.5)^f,^ ^a^	10	(47.6)
We closely follow the extent to which residents can move about independently.	Always^e^	22	(59.5)^a^	8	(50.0)	14	(66.7)^a^
Residents are encouraged to move about independently (e.g., to the living room, the toilet, the activity room).	Always^e^	23	(62.2)^a^	10	(62.5)^a^	13	(61.9)^a^
We instruct residents how they can use walking aids to move about independently.	Always^e^	22	(59.5)^a^	12	(75.0)^a^	10	(47.6)

^a^Floor or ceiling effect present (endorsement frequency of extreme value >50 % of population)

No statistical testing was performed

^b^Mobility-prone group: nursing staff working in a rehabilitation ward or nursing staff specialized in mobility

^c^Non-mobility group: nursing staff not working in a rehabilitation ward and nursing staff not specialized in mobility

^d^Range: completely disagree - completely agree

^e^Range: never – always

^f^Item was completed by a total of 36 respondents

Based on comments of respondents and a last discussion by authors NOK, GARZ, and GJJWB, some final adjustments in wording of items were made and five items that were covered by other items were removed.

### Final inventory

The final version of the MAINtAIN is included in Additional file 1 (the Dutch version of the MAINtAIN is available upon request). MAINtAIN-behaviors, regards the functional activity optimizing behaviors nursing staff can perform and is comprised of 19 items. Each item is rated on a nine-point scale ranging from ‘never’ to ‘always’, labeling the extremes and the middlemost response option. MAINtAIN-barriers, regarding the perceived barriers and facilitators to optimizing functional activity among residents is comprised of 33 items. Again, each item is rated on a nine-point scale, ranging from ‘never’ to ‘always’ for 12 items, and ‘completely disagree’ to ‘completely agree’ for 21 items. The extremes and the middlemost response options are also labeled. A general overview of the nursing behaviors and barriers and facilitators incorporated in the inventory is provided in Table 3.

**Table 3:** Overview of items and concepts in the MAINtAIN

MAINtAIN-behaviors: Nursing behaviors to optimize function					
Items	Nursing behaviors related to type of activity				
Item 1 to 8:	Nursing behavior related to activities of daily living				
Item 9 to 14:	Nursing behaviors related to household activities				
Item 15 to 19:	Nursing behaviors related to miscellaneous activities				
MAINtAIN-barriers: Barriers and facilitators related to behaviors to optimize function, by level					
Item	Residents		Item	Social context	
1.	Relevance for residents	B	19.	Collaboration with experts	B
2.	Capabilities residents	B	20.	Social support of colleagues	F
3.	Visibility of results	F	21.	Support of manager	F
4.	Residents’ fear	B	22.	Referral to responsibility	F
5.	Attention seeking behavior	B	23.	Communication within team	F
6.	Residents’ and families’ expectations regarding care	B	24.	Expectations of colleagues	F
7.	Attitude residents	B	25.	Care routines	B
8.	Learned dependency	B			
Item	Professionals		Item	Organizational & economic context	
9.	Prioritizing time over care	B	26.	Organizational readiness	B
10.	Risks for residents	B	27.	Presence of expertise	F
11.	Task perception: task of physiotherapist	B	28.	Educational opportunities	F
12.	Sense of importance	F	29.	Rules and regulations	B
13.	Task perception: taking responsibility	F	30.	Availability of resources	F
14.	Outcome expectations	B	31.	Priority within organization	F
15.	Availability of expertise	F	32.	Staffing level	B
16.	Conflict: time consuming	B	33.	Time	F
17.	Sense of difficulty	B			
18.	Self-efficacy	F			

*B* item formulated as barrier

*F* item formulated as facilitator

Levels of barriers and facilitators are derived from Grol and Wensing [13]

Additional file 1 includes the MAINtAIN

## Discussion

In a step-wise comprehensive mixed-methods study, involving a multidisciplinary group of stakeholders, MAastrIcht Nurses Activities INventory (MAINtAIN), was developed. The MAINtAIN gains insight into the extent to which nursing staff perceive to perform behaviors to optimize functional activity among nursing home residents, and their perceived barriers and facilitators related to this behavior. It assesses nursing staff behavior during a broad range of activities, i.e., ADL, household activities and miscellaneous activities. Additionally, it addresses barriers and facilitators related to different levels, i.e., the residents, the professionals, the social context, and the organizational and economic context. A cross-sectional study showed that the MAINtAIN is a usable inventory to collect data on these topics.

The comprehensive development process of the MAINtAIN (including various strategies, e.g., literature searches, expert consultations, interviews with a heterogeneous sample of individuals) resulted in an inventory including behaviors, and barriers and facilitators that are considered important by the stakeholders. Although nursing staff will be the end users of the MAINtAIN, the heterogeneous sample of experts involved in the study warrants the inclusion of important behaviors, barriers and facilitators. According to different experts the selected nursing behaviors are both relevant and feasible, and together they provide an overview of the extent to which nursing staff perceive to perform functional activity-optimizing behaviors. In addition, the broad range of barriers and facilitators selected here are relevant to nursing staff, covering topics on several levels, from factors relating to residents to factors relating to the organizational context. Mapping such a variety of factors is important in order to address factors to improve care [24].

Although the MAINtAIN was developed in the Netherlands, the incorporated nursing behaviors, barriers and facilitators are generalizable to other countries with different healthcare systems as international literature was used to develop the inventory. Barriers and facilitators included in the MAINtAIN overlap with those described in previous research regarding function focused care [12, 20]. In addition, included barriers and facilitators correspond with barriers and facilitators mentioned in international studies of evidence-based practice or innovations in general [25]. Nonetheless, nursing home populations can differ within and between countries. To be able to interpret and compare results from different studies that use the MAINtAIN, carefully describing populations within studies is recommended.

To our knowledge there are no other inventories or questionnaires available that measure the extent to which nursing staff report to provide functional activity optimizing care, as MAINtAIN-behaviors does. Although studies indicate that nursing home residents are largely inactive [4–6], and participation in household activities is low [3, 6], there are no studies that provide insight into the behaviors nursing staff generally perform to encourage the broad range of activities in which nursing home residents can play an active role. Resnick et al. [17] developed and applied an observational checklist to measure an approach to improve function. This instrument assesses ADL activities and exercise. In the development process of the current study, this checklist, as well as various other sources, was considered. This provided valuable information and resulted in the inclusion of a broader range of items on nursing behaviors. MAINtAIN-behaviors, for example, also includes the encouragement of household activities. Although direct observations provide valuable information, the advantages of the MAINtAIN are that it is easy and quick for nursing staff to use and applicable on a large scale. Furthermore, it takes into account the perceptions of the nursing staff. Quality improvement projects could use a combination of direct observations and the MAINtAIN-behaviors to reveal possible misperceptions among nursing staff. With regard to MAINtAIN-barriers, there are no other inventories that measure barriers and facilitators tailored to this specific problem and this specific setting. This is significant since literature shows that barriers and facilitators are setting-specific and related to the addressed problem [26]. The MAINtAIN fills this gap.

### Limitations

There are some limitations to this study that should be addressed. First, this article focuses on the development process of the MAINtAIN. Although content validity was assessed, it is recommended that future studies extensively investigate multiple psychometric properties of both parts of the MAINtAIN. Such a study could address the MAINtAIN’s construct validity, reliability, and its sensitivity to change. For MAINtAIN-behaviors the internal consistency of the items related to ADL, household activities, and miscellaneous activities can be determined. Due to the small sample size of our usability study (*N* = 37), this was not possible in the present study. Second, although research shows that social desirability bias does not, by definition, have to occur [27], nursing staff may consciously or subconsciously present themselves or their ward in a positive light. This may be particularly important for MAINtAIN-behaviors. However, in the development process of the inventory, actions were taken to prevent social desirability, e.g., by formulating items on a less individual level. In addition, the anonymous administration of the inventory is recommended to prevent social desirable responses. Moreover, the MAINtAIN assesses self-reported behavior and barriers and facilitators as perceived by the nursing staff; it provides information on perceptions that may or may not accurately reflect the actual situation. Users of the inventory need to keep this in mind when they use and interpret the results of the MAINtAIN.

### Implications for research and practice

The next step is to administer the MAINtAIN on a large scale. Not only would this make it possible to investigate its psychometric properties, the information obtained with the MAINtAIN would provide researchers and practitioners opportunities to improve nursing care. Based on this information, strategies can be developed to promote nursing behavior, tackle specific barriers, and promote certain facilitators. This could lead to an improvement in nursing behaviors and eventually lead to optimized or maintained functional activity among nursing home residents.

Furthermore, the MAINtAIN offers nursing homes the possibility to see where their priorities lie, what kind of nursing behaviors are performed according to the nursing staff, and to see what barriers and facilitators are particularly relevant for their nursing home. The outcomes of the current study showed that the MAINtAIN is easy to use in daily practice. Nursing staff themselves can assess factors that have to be addressed in order to improve care.

## Conclusion

The MAINtAIN is a usable method for researchers and nursing staff to obtain insight into the perceived behaviors nursing staff perform to optimize and maintain functional activity among residents. In addition, it maps nursing staff perceived barriers and facilitators to this behavior. Although further psychometric research is recommended, the present inventory provides researchers and practitioners with the opportunity to gain insight into these factors and eventually an opportunity to improve care in nursing homes.

## Additional file

Additional file 1:
**the MAINtAIN.** MAINtAIN-behaviors and MAINtAIN-barriers are included in Additional file 1. (PDF 628 kb)

## Abbreviations

ADL: activities of daily living
CNA: certified nurse assistant
MAINtAIN: MAastrIcht Nurses Activities INventory
RN: registered nurse

## Footnotes

1 In this article nurses with three years of secondary vocational training are called CNAs, they are comparable to Licensed Practical Nurses in the United States [28]. CNAs form the major workforce in nursing homes in the Netherlands.
