# Supplementary material for: Development and usability of the MAINtAIN, an inventory assessing nursing staff behavior to optimize and maintain functional activity among nursing home residents: a mixed-methods approach
Source: BMC Health Serv Res. 2016 Feb 2;16:38. doi: 10.1186/s12913-016-1288-7 (PMC4736173; doi:10.1186/s12913-016-1288-7)
Supplement: Additional file 1: — the MAINtAIN. MAINtAIN-behaviors and MAINtAIN-barriers are included in Additional file 1. (PDF 628 kb) [file 12913_2016_1288_MOESM1_ESM.pdf]

Additional file 1. The MAINtAIN

Below is the English version of the MAINtAIN. The MAINtAIN was translated from Dutch to English using forward-backward translation procedures [1].The translation procedure consisted of three phases. First, the MAINtAIN was translated into English by two bilingual independent translators. One of them had a nursing background; the other was a professional translator. Differences were discussed and from these two translations one version was created. Second, this version was translated back into Dutch by two other independent bilingual translators. Consensus was reached on one final Dutch translation. Lastly, the authors (NOK, GARZ, GJJWB) checked the accuracy of the English translation, by comparing all translations.

1. Beaton DE, Bombardier C, Guillemin F, Ferraz MB. Guidelines for the process of cross-cultural adaptation of self-report measures. Spine. 2000;25(24):3186-91.

This research project was funded by: ZonMw

MAINtAIN-behaviors: Activity in the care for resident

Please indicate for each of the following questions which answer best describes how things work in your ward. When answering these questions, consider the residents for whom this is relevant; the questions are not applicable for completely paralyzed or terminal residents. Only cross one circle for each question.

On my ward…

	**ADLs in general (multiple aspects)**	*Never*				*Some-times*				*Always*
1.	...we closely follow the extent to which residents are able to perform ADLs independently.	○	○	○	○	○	○	○	○	○
2.	.... we encourage residents to perform ADLs independently as much as possible.	○	○	○	○	○	○	○	○	○
	**Eating**	*Never*				*Some-times*				*Always*
3.	...we discuss with residents* whether they need assistive devices to help them eat.	○	○	○	○	○	○	○	○	○
	**Dressing**	*Never*				*Some-times*				*Always*
4.	...we compliment residents when they dress and undress themselves.	○	○	○	○	○	○	○	○	○
5.	... we discuss with residents which assistive devices they need in order to get dressed as independently as possible.	○	○	○	○	○	○	○	○	○
	**Moving about**	*Never*				*Some-times*				*Always*
6.	...we closely follow the extent to which residents can move about independently.	○	○	○	○	○	○	○	○	○
7.	…residents are encouraged to move about independently (e.g., to the living room, the toilet, the provided activities).	○	○	○	○	○	○	○	○	○
	**Personal hygiene**	*Never*				*Some-times*				*Always*
8.	…we lay out the assistive devices that are needed for bathing/showering so that residents can bath themselves as independently as possible.	○	○	○	○	○	○	○	○	○

**If residents cannot communicate this themselves, then this can read: residents and/or family*

Nienke O. Kuk, G.A. Rixt Zijlstra, Gerrie J.J.W. Bours. Jan P.H. Hamers, Gertrudis I.J.M. Kempen

Maastricht University

For the following questions, consider residents for whom this is relevant: the questions are not applicable for completely paralyzed or terminal residents.

On my ward…

	**Household activities**	*Never*				*Some-times*				*Always*
9.	…we prepare sandwiches for residents, even if they can do this themselves.	○	○	○	○	○	○	○	○	○
10.	...we encourage residents to help set and clear the table.	○	○	○	○	○	○	○	○	○
11.	…we make the beds, even if the residents can do this (partly) themselves.	○	○	○	○	○	○	○	○	○
12.	...we encourage residents to fold or put away their own clothes.	○	○	○	○	○	○	○	○	○
13.	…we encourage residents to do light household chores (e.g., cleaning the bedroom or living room, cleaning the placemats, washing the dishes).	○	○	○	○	○	○	○	○	○
14.	...we discuss with residents* which household chores they can help with.	○	○	○	○	○	○	○	○	○
	**Miscellaneous activities**	*Never*				*Some-times*				*Always*
15.	…we encourage residents to participate in organized activities in which they are physically active (such as walking, (wheelchair) dancing).	○	○	○	○	○	○	○	○	○
16.	…we discuss with residents* what kind of activities (ADLs, household activities and social activities) they used to do and we try to maintain them.	○	○	○	○	○	○	○	○	○
17.	…we encourage family/informal caregivers only to help residents when they cannot do something themselves.	○	○	○	○	○	○	○	○	○
18.	...encouraging physical activity is part of the care plan.	○	○	○	○	○	○	○	○	○
19.	…we discuss with residents which ADLs and household activities they would like to perform.	○	○	○	○	○	○	○	○	○

**If residents cannot give a good indication of this themselves, this can read: residents and/or family*

Nienke O. Kuk, G.A. Rixt Zijlstra, Gerrie J.J.W. Bours. Jan P.H. Hamers, Gertrudis I.J.M. Kempen

Maastricht University

MAINtAIN-barriers: Encouraging activity - underlying factors

In the following statements, encouraging activity under residents is described. The terms 'physical activity' or 'activities' are used to describe the activities of daily living (ADL) or household activities performed by residents.

ADL: activities such as bathing, dressing, toileting, moving about the ward and eating

Household activities: activities such as preparing breakfast and lunch, cleaning, setting the table and washing the dishes.

Please indicate for each of the following questions or statements which answer best describes how things generally work on your ward. Only cross one circle for each question. There are no right or wrong answers; we are asking about your opinion.

Factors concerning residents and their families: characteristics that influence your work

	Completely disagree				*Neither agree, nor disagree*				*Completely agree*
1. It is not relevant for residents on my ward to independently perform ADLs (e.g., bathing and dressing).	○	○	○	○	○	○	○	○	○
2. Residents on my ward are not able to perform ADLs more independently than they currently do.	○	○	○	○	○	○	○	○	○
3. I see that encouraging physical activity has a positive effect on the residents.	○	○	○	○	○	○	○	○	○

	Never				*Some-times*				*Always*
4. Residents are afraid to walk on their own.	○	○	○	○	○	○	○	○	○
5. Residents ask for help with ADLs (e.g., eating and bathing) so that they can get extra attention.	○	○	○	○	○	○	○	○	○
6. Residents and/or family expect the nursing staff to take over the activities that the residents themselves can still perform.	○	○	○	○	○	○	○	○	○
7. Residents do not want to perform activities themselves (such as bathing, move about in a wheelchair), even if they still can.	○	○	○	○	○	○	○	○	○
8. Residents on my ward consider it perfectly normal to have others move them instead of moving about themselves.	○	○	○	○	○	○	○	○	○

Nienke O. Kuk, G.A. Rixt Zijlstra, Gerrie J.J.W. Bours. Jan P.H. Hamers, Gertrudis I.J.M. Kempen

Maastricht University

Physical activity & activities
ADL: activities such as bathing, dressing, toileting, moving about the ward and eating
Household activities: activities such as preparing breakfast and lunch, cleaning, setting the table and washing the dishes

Factors concerning the professionals: what you and your colleagues experience

	Never				*Some-times*				*Always*
9. On my ward, we consider organizing our work so that residents are ready on time more important than allowing them to independently perform their ADLs (e.g., eating, bathing).	○	○	○	○	○	○	○	○	○
10. I am afraid that residents will hurt themselves if I encourage them to walk alone.	○	○	○	○	○	○	○	○	○

	Completely disagree				*Neither agree, nor disagree*				*Completely agree*
11. It is primarily the responsibility of the physiotherapist/occupational therapist to encourage residents to perform activities.	○	○	○	○	○	○	○	○	○
12. On my ward, we think that is important to encourage residents to perform ADLs (e.g., moving about, eating, bathing) as independently as possible.	○	○	○	○	○	○	○	○	○
13. On my ward, we consider it our responsibility to inform the family/informal caregivers about the importance of residents performing activities independently.	○	○	○	○	○	○	○	○	○
14. I expect that encouraging ADLs and household activities has no effect on how residents function.	○	○	○	○	○	○	○	○	○
15. On my ward, sufficient expertise is available to encourage residents to be as independent as possible in performing ADLs (e.g., bathing and moving about).	○	○	○	○	○	○	○	○	○
16. Encouraging residents to perform ADLs as independently as possible gives me less time for other things.	○	○	○	○	○	○	○	○	○
17. I find it difficult to encourage residents to be more active.	○	○	○	○	○	○	○	○	○
18. I f I want, I am able to allow residents to perform ADLs (e.g., eating and bathing) more independently.	○	○	○	○	○	○	○	○	○

Nienke O. Kuk, G.A. Rixt Zijlstra, Gerrie J.J.W. Bours. Jan P.H. Hamers, Gertrudis I.J.M. Kempen

Maastricht University

Physical activity & activities
ADL: activities such as bathing, dressing, toileting, moving about the ward and eating
Household activities: activities such as preparing breakfast and lunch, cleaning, setting the table and washing the dishes.

Factors concerning the social environment: how the team functions

	Completely disagree				*Neither agree, nor disagree*				*Completely agree*
19. On my ward, collaboration with experts is not good enough (for example an occupational or physical therapist) to support residents in performing their ADLs as independently as possible.	○	○	○	○	○	○	○	○	○
20. I can count on enough support from my colleagues if I allow residents to perform ADLs and household activities as independently as possible.	○	○	○	○	○	○	○	○	○

	Never				*Some-times*				*Always*
21. My direct manager communicates that it is important to encourage residents to be physically active and to perform ADLs and household activities as independently as possible.	○	○	○	○	○	○	○	○	○
22. I speak to my colleagues if I see them doing activities that residents can still perform themselves.	○	○	○	○	○	○	○	○	○
23. We discuss within the team how we can encourage residents to perform their ADLs and household activities as independently as possible.	○	○	○	○	○	○	○	○	○
24. My colleagues expect me to encourage residents to help carry out household activities.	○	○	○	○	○	○	○	○	○
25. On my ward, it is our routine to take over the ADLs and household tasks (e.g., making sandwiches) for the residents.	○	○	○	○	○	○	○	○	○

Physical activity & activities
ADL: activities such as bathing, dressing, toileting, moving about the ward and eating
Household activities: activities such as preparing breakfast and lunch, cleaning, setting the table and washing the dishes.

Factors concerning your organization: how things work in your nursing home

	Completely disagree				*Neither agree, nor disagree*				*Completely agree*
26. My organization is not geared towards involving residents in doing household activities (such as preparing meals and cleaning rooms).	○	○	○	○	○	○	○	○	○
27. There are enough people in my organization with knowledge about how to encourage residents to be physically active and perform activities independently.	○	○	○	○	○	○	○	○	○
28. My organization offers the possibility to attend internal or external courses that address how to encourage physical activity among residents.	○	○	○	○	○	○	○	○	○
29. My organization has no agreements or guidelines on how to encourage residents to be physically active.	○	○	○	○	○	○	○	○	○
30. The organization has sufficient resources (e.g., funding or facilities) to enable nursing staff to encourage residents to be physically active.	○	○	○	○	○	○	○	○	○
31. In my organization, encouraging physical activity is a high priority.	○	○	○	○	○	○	○	○	○
32. There is a structural shortage of staff available to try to encourage residents to perform ADLs (e.g., eating and bathing) as independently as possible.	○	○	○	○	○	○	○	○	○
33. There is sufficient time to allow residents to perform ADLs themselves (e.g., bathing, eating, and dressing).	○	○	○	○	○	○	○	○	○

Nienke O. Kuk, G.A. Rixt Zijlstra, Gerrie J.J.W. Bours. Jan P.H. Hamers, Gertrudis I.J.M. Kempen
